# Prediction model for successful induction of labor by cervical strain elastography diagnosed at late-term pregnancy in nulliparous women: a prospective cohort study

**DOI:** 10.1186/s12884-023-05426-7

**Published:** 2023-02-14

**Authors:** Qing Yang, Chen-Chen Zhou, Ying Chen, Jin-Dan Pei, Xiao-Lin Hua, Li-Ping Yao

**Affiliations:** 1 Department of Ultrasound, Shanghai Key Laboratory of Maternal Fetal Medicine, Shanghai Institute of Maternal-Fetal Medicine and Gynecologic Oncology, Shanghai First Maternity and Infant Hospital, School of Medicine, Tongji University, Shanghai, 200092 China; 2 Department of Obstetrics, Shanghai Key Laboratory of Maternal Fetal Medicine, Shanghai Institute of Maternal-Fetal Medicine and Gynecologic Oncology, Shanghai First Maternity and Infant Hospital, School of Medicine, Tongji University, Shanghai, 200092 China

**Keywords:** Prediction model, Labor induction, Cervical strain elastography, Nulliparous, Women, Late-term pregnancy

## Abstract

**Background:**

The use of cervical strain elastography for nulliparous women during late-term pregnancy remains unclear. This study assesses the predictive value of late-term cervical strain elastography for successful induction of labor (IOL) in nulliparous women.

**Methods:**

This single-centered, prospective study included 86 patients undergoing IOL between January 2020 and March 2022. Univariate and multivariate analyses were conducted to identify predictive factors for successful IOL. The predictive values were assessed using the area under receiver operating characteristic (ROC) curves.

**Results:**

IOL was successful in 58 patients. The hardness ratio and cervical length were significantly associated with successful late-term IOL in nulliparous women. The predictive value of the combination of hardness ratio and cervical length was higher than that of cervical length alone.

**Conclusions:**

The hardness ratio and cervical length assessed by cervical strain elastography during late-term pregnancy are predictors of the success of IOL in nulliparous women. The predictive value of the combination of hardness ratio and cervical length was higher than that of cervical length alone.

## Background

Induction of labor (IOL) is the artificial initiation of the labor process before its spontaneous onset to achieve a vaginal delivery [1]. IOL is necessary in 18.4–33.3% of women and the prevalence of IOL is higher in developed countries than in developing countries [2, 3]. The risk for mothers and fetuses is increased during the third trimester, and the risks of adverse pregnancy outcomes are elevated at 41^+6^ weeks of gestation. Therefore, IOL is recommended for pregnancies over 41 weeks of gestation without spontaneous parturition [4, 5]. However, unsuccessful IOL occurs in 17.3–36.8% of women [6, 7] and is associated with an increased risk of cesarean section and postpartum and neonatal complications [8].

Cervical status, parity, body mass index (BMI), gestational age, and estimated fetal weight affect the success of IOL [9, 10]. The most important predictor for successful IOL is the pre-induction cervical status. Several technologies are available to assess the cervical condition. The onset of labor is predicted using the Bishop score (BS) or cervical length to assess the cervix. The BS is used to classify the cervix as “favorable” or “unfavorable,” though the examination is subjective and causes the patient discomfort [11, 12]. Therefore, new techniques for the assessment of the cervix prior to IOL are needed.

Several studies have used transvaginal ultrasound to measure the cervical length and predict the success of IOL [13–15]. These previous studies reported that a longer cervical length is associated with an increased risk of unsuccessful IOL. Ultrasound elastography can be used to assess the biochemical and mechanical properties of the cervical tissue [16, 17]. The stiffness of the cervical tissue can be assessed using strain elastography via intrinsic compression. The entire cervix can be evaluated as the region of interest (ROI) and the heterogeneity of the entire cervix can be analyzed [18]. However, the clinical usefulness of cervical strain elastography is limited by the lack of standardized measurements. ElastoScan for the cervix (E-cervix system) was created to overcome the limitations of ultrasound elastography. The E-cervix system can be used to measure several parameters related to cervical stiffness via tissue displacement induced by physiological arterial pulsations. The E-cervix system has acceptable reproducibility in pregnant women at different stages of pregnancy and is considered an important diagnostic tool for assessing cervical strain [19]. However, most previous studies used the E-cervix system to evaluate cervical length only, and the predictive values of other cervical strain elastography parameters have not been reported. Therefore, this study assesses the reproducibility and predictive value of cervical strain elastography parameters for successful IOL in nulliparous women during late-term pregnancy.

## Methods

### Patients

This single-centered prospective, observational study included women pregnant with singletons at 41–41^+6^ weeks of gestation who visited the antenatal outpatient department at Shanghai First Maternity and Infant Hospital affiliated with Tongji University for a late-term check-up from January 2020 to March 2022. The gestational age was determined using ultrasound fetometry at 12–20 weeks of gestation. Inclusion criteria were as follows: (1) gestational age at 41–41^+6^ weeks; (2) BS between 4–6, with intact amniotic membranes and no placenta previa or vaginal bleeding on ultrasound examination; (3) live fetus with a cephalic presentation. The exclusion criteria were as follows: (1) Multiparous women, those with a history of cervical insufficiency or cervical surgery, and those with hypertension or diabetes; (2) Patients with fetal abnormalities or contraindications for vaginal delivery. This study was approved by the Institutional Review Board of Shanghai First Maternity and Infant Hospital (approval number KS20186), and all patients provided written informed consent.

### Data collection

The maternal weight and height were measured at the time of hospital admission. Maternal characteristics and obstetric history data were extracted from the hospital medical system database. The BS was evaluated by clinical obstetricians with at least ten years of experience in the obstetric field, and the examiners were not aware of the patients’ enrollment in the study. The pregnancy and neonate outcome data were retrieved from the electronic medical records.

### Cervical elastography

**Fig. 1:**
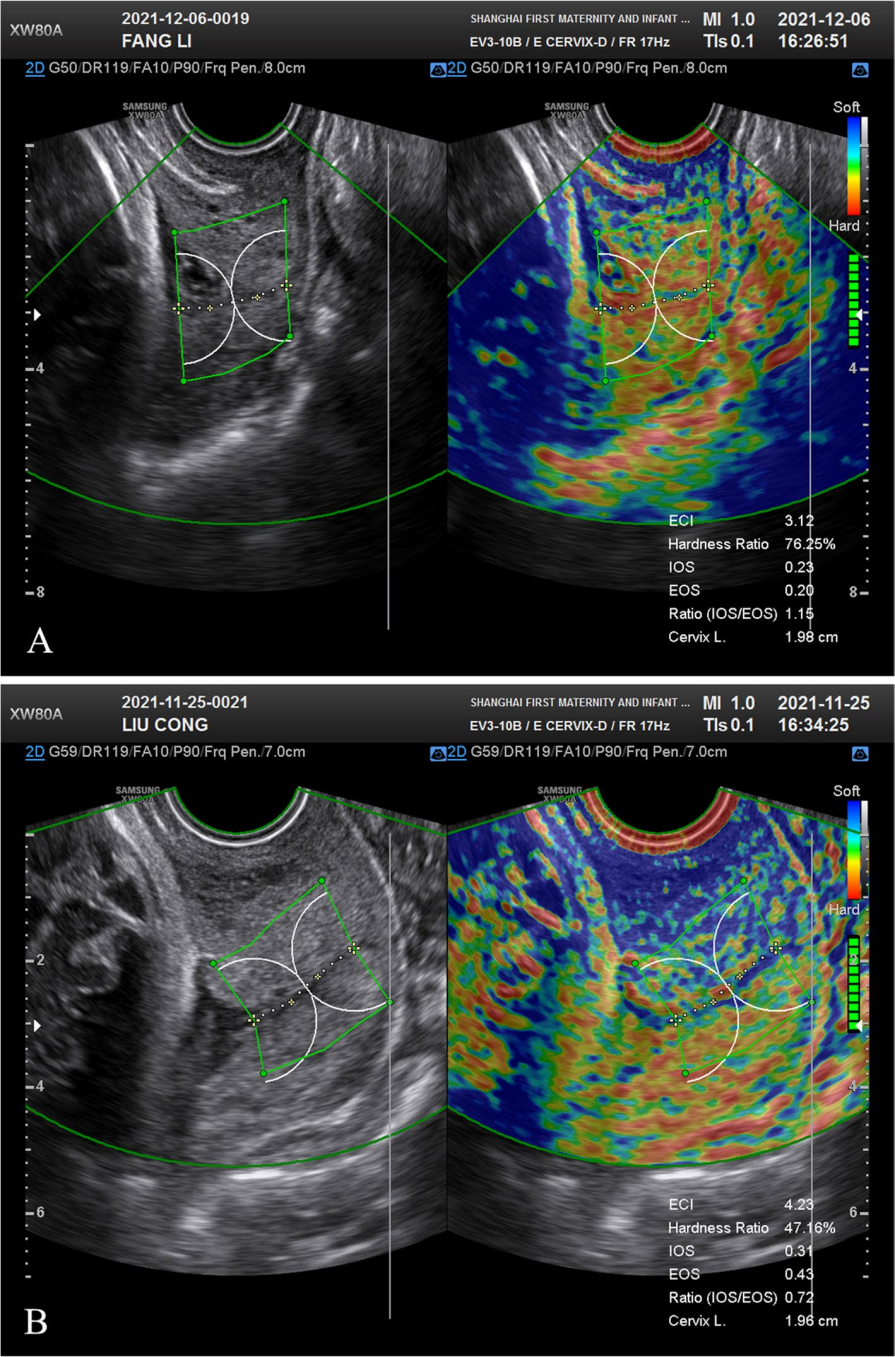
The cervical strain elastography assessments at 41 weeks in two patients with similar cervical length (A, 19.8 mm; B, 19.6 mm) but different hardness ratio values (A, 76.25%; B, 47.16%) are shown. The patient in panel A underwent a cesarean delivery after 24 h. The patient in panel B had a vaginal delivery after 11.83 h

A Samsung WS80A ultrasound device (Samsung Medison, Seoul, South Korea) with a 6 MHz vaginal ultrasound transducer and the E-cervix system were used. After emptying their bladder, the patients were instructed to lay in the dorsal lithotomy position. The transducer was gently inserted and placed at the anterior fornix of the vagina. Using the E-cervix program, the operator conducted cervical strain elastography with dual images, including a grayscale image on the left and an elastogram on the right side of the screen (Fig. 1). No additional external pressure was applied to the cervix by the operator, and the patient was asked to breathe normally. The sonographers were blinded to the BS.

The elastographic parameters were calculated using the E-cervix system. The operator drew the cervical canal and used a four-point ROI to trace the endocervical lining between the internal os (IOS) and external os (EOS) of the cervix. After the cervical canal was defined, the ROI that included the entire cervical area automatically appeared. The operator manually redefined the ROI as necessary. Two fan-shaped ROIs with radii of 1 cm were automatically generated around the IOS and EOS. The E-cervix system was used to calculate the elasticity index (ECI), a measurement of tissue heterogeneity defined as the average contrast index of the pixels. The ECI ranged from 0 (homogeneous) to 81 (heterogeneous). The hardness ratio (HR) was also calculated using the E-cervix system. The HR was defined as the number of red pixels (the top 30% of the color intensity scale) divided by the total number of pixels in the ROI. The HR ranged from 0% (soft) to 100% (hard). The mean average strain values of the IOS and EOS ranged from 0 (hard) to 1 (soft) and were also calculated using the E-cervix system. The IOS/EOS mean strain ratio and cervical length were also determined. When funneling was present, the residual cervical length was measured and the funneling was excluded from the measurement, yielding the functional cervical length [20].

### Reproducibility of cervical length and elastography

To assess the interobserver variability, two specialists independently performed the E-cervix examination on the first 40 patients according to a standard protocol. The results were blinded to the two specialists. One ultrasound specialist performed the examination twice for 40 patients (selected randomly) to assess the intraobserver variability.

### Induction of labor

Pregnant women with a BS of 6 underwent artificial rupture of the membranes 6 h after admission. Patients with a BS < 6 points without leukorrhea were induced using a Cook cervical ripening balloon (Cook Incorporated, Bloomington, IN, USA). The balloon was removed after 12 h, and the BS was evaluated again. Patients with a BS ≥ 6 underwent artificial rupture of the membranes, and patients with a BS < 6 were administered intravenous micro-pump oxytocin. Patients who did not have spontaneous uterine contractions within 2 h of artificial rupture of the membranes were administered intravenous micro-pump oxytocin.

The investigated outcome was successful IOL, which was defined as active labor within 9 h or delivery within 24 h [21], and active labor was defined as a cervical dilation of at least 6 cm [22]. Failure to enter the active phase was defined as the failure of the cervix to efface and dilate to 3 cm within 12 h after the artificial rupture of the membranes, the initiation of oxytocin, or a BS < 6 at 24 h [23].

### Statistical analysis

Continuous variables are presented as mean (standard deviation) and median (interquartile range) according to the data distribution, while categorical variables are presented as frequencies and proportions. The differences between the groups were assessed using the t-test or non-parametric test for continuous variables and the chi-square test for categorical variables. The interclass correlation coefficient (ICC) was used to assess the intra- and interobserver reproducibility of elastographic parameters. The ICC was classified as poor reproducibility (< 0.50), moderate reproducibility (0.50–0.75), good reproducibility (0.75–0.90), or excellent reproducibility (> 0.90). Univariate and multivariate logistic regression analyses were performed to identify the predictors of successful IOL, while receiver operating characteristic (ROC) curves were created for each parameter to evaluate the predicted performance. The area under the ROC curve (AUC) and 95% confidence interval (CI) were applied to the predictive value. The optimal cutoff was determined using the Youden index, and the sensitivity, specificity, positive and negative predictive values, and AUC were calculated. The AUCs were compared using the DeLong test. All P-values were two-sided, and *P* < 0.05 was considered statistically significant. All statistical analyses were conducted using Statistical Package for Social Sciences software (version 24.0; SPSS Inc., Chicago, IL, USA).

## Results

### Patient characteristics

**Fig. 2:**
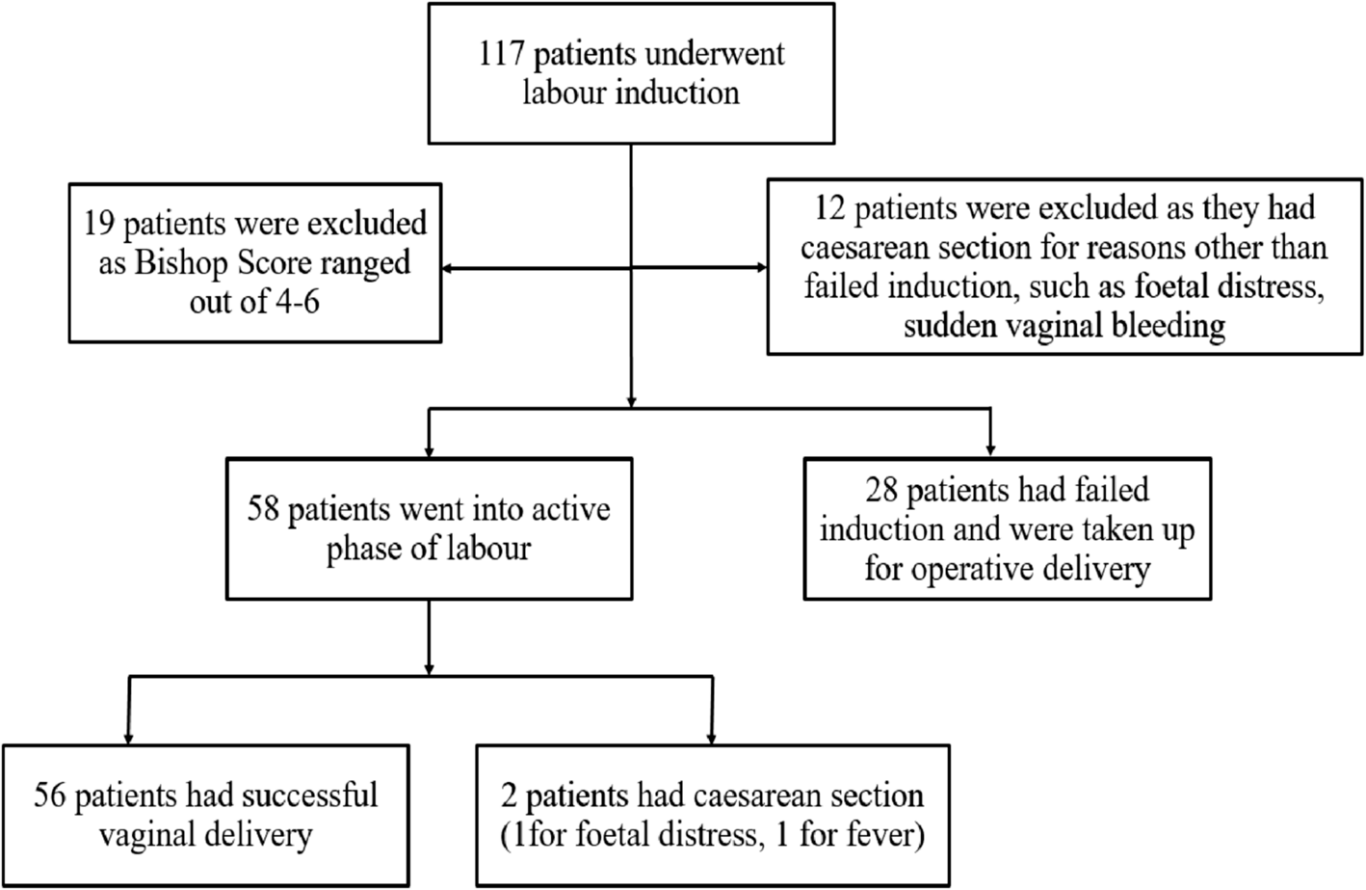
Patient flow chart

During the study period, 117 patients were considered eligible, though 31 were excluded due to a cesarean delivery for fetal distress (*n* = 5), vaginal bleeding (*n* = 7), and a BS < 4 or > 6 (*n* = 19). The remaining 86 patients were included in the final analyses (Fig. 2). IOL was successful in 58 patients (successful IOL group, 67.44%) and unsuccessful in 28 patients (unsuccessful IOL group, 32.56%) who ultimately underwent cesarean delivery. There were no significant differences in maternal age (*P* = 0.224), pre-pregnancy BMI (*P* = 0.231), gestational age at IOL (*P* = 0.074), BS (*P* = 1.000), ECI (*P* = 0.627), IOS (*P* = 0.414), EOS (*P* = 0.757), IOS/EOS ratio (*P* = 0.712), neonatal birth weight (*P* = 0.389), Apgar score (after 5 min) (*P* = 0.236), neonatal sex (*P* = 0.066), or postpartum hemorrhage (*P* = 0.306) between the groups (Table 1). The cervical length (*P* = 0.010), HR (*P* < 0.001), gestational age at delivery (*P* = 0.006), and Apgar scores after 1 min (*P* = 0.006) were significantly different between the groups.

**Table 1:** Maternal and neonatal characteristics of enrolled participants

	Total (*n* = 86)	Successful IOL (*n* = 58)	Unsuccessful IOL (*n* = 28)	*P* value
Maternal age (years)	30.47 ± 3.62	30.80 ± 3.73	29.70 ± 3.30	0.224
Pre-pregnancy, BMI(kg/m^2^)	25.62 ± 1.35	25.50 ± 1.28	25.88 ± 1.48	0.231
GA at IOL (week)	41.13 ± 0.20	41.16 ± 0.22	41.06 ± 0.09	0.074
Bishop score	4.00 (4.00,4.00)	4.00 (4.00,4.00)	4.00 (4.00,4.00)	1.000
Cervical length (mm)	25.40 (16.65,31.52)	21.53 (14.70,30.96)	27.63 (22.50,31.93)	0.010*
ECI	3.65 ± 1.45	3.70 ± 1.36	3.54 ± 1.62	0.627
HR	49.81 (36.11,58.20)	44.40 (32.57,52.01)	58.21 (49.78,61.93)	0.000*
IOS	0.38 ± 0.09	0.39 ± 0.09	0.36 ± 0.10	0.414
EOS	0.39 ± 0.11	0.39 ± 0.09	0.38 ± 0.16	0.757
IOS / EOS Ratio	1.01 ± 0.23	1.02 ± 0.23	1.00 ± 0.24	0.712
GA at delivery (weeks)	42.09 ± 0.35	42.00 ± 0.33	42.33 ± 0.25	0.006*
Neonatal birth weight (g)	3371.78 ± 267.32	3356.49 ± 275.97	3431.67 ± 231.06	0.389
Apgar after 1 min	9 (9,10)	9 (9,10)	9 (9,9)	0.006*
Apgar after 5 min	10 (10,10)	10 (10,10)	10 (9,10)	0.236
Neonatal Sex				0.066
Male	37 (43.02%)	21(36.21%)	16 (57.14%)	
Female	49 (56.98%)	37(63.79%)	12 (42.86%)	
Hemorrhage of deliver (mL)	348.56 ± 104.21	355.64 ± 114.03	320.83 ± 39.65	0.306

*BMI *Body mass index, *GA *Gestational age, *IOL* Induction of labor, *ECI* Elasticity contrast index, *HR* Hardness ratio, *IOS* Mean strain level of the internal os, *EOS* mean strain level of the external os

^*^ *P* < 0.05

### Intra- and interobserver reproducibility analysis

The ICCs for ECI (ICC: 0.871; 95% CI: 0.760–0.941), HR (ICC: 0.839; 95% CI: 0.732–0.905), and EOS (ICC: 0.777; 95% CI: 0.648–0.865) suggested good intraobserver reproducibility, while the ICCs for IOS (ICC: 0.709; 95% CI: 0.537–0.842) and IOS/EOS ratio (ICC: 0.672; 95% CI: 0.527–0.779) suggested moderate intraobserver reproducibility (Table 2). The ECI (ICC:0.777; 95% CI: 0.636–0.874) had good interobserver reproducibility, while HR (ICC: 0.677; 95% CI: 0.469–0.814), IOS (ICC: 0.676; 95% CI: 0.508–0.805), EOS (ICC: 0.639; 95% CI: 0.449–0.771), and IOS/EOS ratio (ICC: 0.521; 95% CI: 0.372–0.666) had moderate interobserver reproducibility (Table 2). The functional cervical length had excellent intraobserver (ICC: 0.953; 95% CI: 0.924–0.973) and interobserver (ICC: 0.949; 95% CI: 0.920–0.971) reproducibility.

**Table 2:** Intra-observer and inter-observer reproducibility for cervical length and all elastographic parameters

Parameters	intra -observer		inter -observer	
	ICC (95%CI)	*P* value	ICC (95%CI)	*P* value
ECI	0.871 (0.760–0.941)	0.000*	0.777 (0.636–0.874)	0.000*
HR	0.839 (0.732–0.905)	0.000*	0.677 (0.469–0.814)	0.000*
IOS	0.709 (0.537–0.842)	0.000*	0.676 (0.508–0.805)	0.000*
EOS	0.777 (0.648–0.865)	0.000*	0.639 (0.449–0.771)	0.000*
IOS/EOS Ratio	0.672 (0.527–0.779)	0.000*	0.521 (0.372–0.666)	0.000*
Cervical Length	0.953 (0.924–0.973)	0.000*	0.949 (0.920–0.971)	0.000*

*ICC* Interclass correlation coefficient, *CI* Confidence interval, *ECI* Elasticity contrast index, *HR* Hardness ratio, *IOS* Internal os, *EOS* External os

^*^ *P* < 0.05

### Predictors of successful IOL

HR (OR: 1.09; 95% CI: 1.03–1.15; *P* = 0.002) and cervical length (OR: 1.08; 95% CI: 1.02–1.14; *P* = 0.011) were significantly associated with the incidence of successful IOL, while ECI, IOS, EOS, and IOS/EOS ratio were not predictors of the success of IOL (Table 3). HR (OR: 1.21; 95% CI: 1.09–1.35; *P* = 0.039) and cervical length (OR: 1.10; 95% CI: 1.02–1.19; *P* = 0.017) were found to be independent predictors of the success of IOL (Table 3).

**Table 3:** Univariate analysis and multivariate analysis of elastographic parameters and cervical length for the incidence of successful IOL

Variables	Univariate OR and 95% CI	*P* value	Multivariate OR and 95% CI	*P* value
ECI	0.92 (0.67–1.27)	0.622	1.37 (0.78–2.41)	0.271
HR	1.09 (1.03–1.15)	0.002*	1.21 (1.09–1.35)	0.039*
IOS	0.12 (0.01–17.73)	0.410	927.23 (0.00–5.11E13)	0.648
EOS	0.52 (0.01–31.51)	0.754	43.31 (0.00–1.089E9)	0.665
IOS / EOS Ratio	1.05 (0.12–9.26)	0.962	23.95 (0.02–353.41)	0.518
Cervical Length	1.08 (1.02–1.14)	0.011*	1.10 (1.02–1.19)	0.017^*^

*OR* Odds ratio, *CI* Confidence interval, *ECI* Elasticity contrast index, *HR* Hardness Ratio, *IOS* Internal os, *EOS* External os

^*^ *P* < 0.05

### Predictive model for successful IOL

**Fig. 3:**
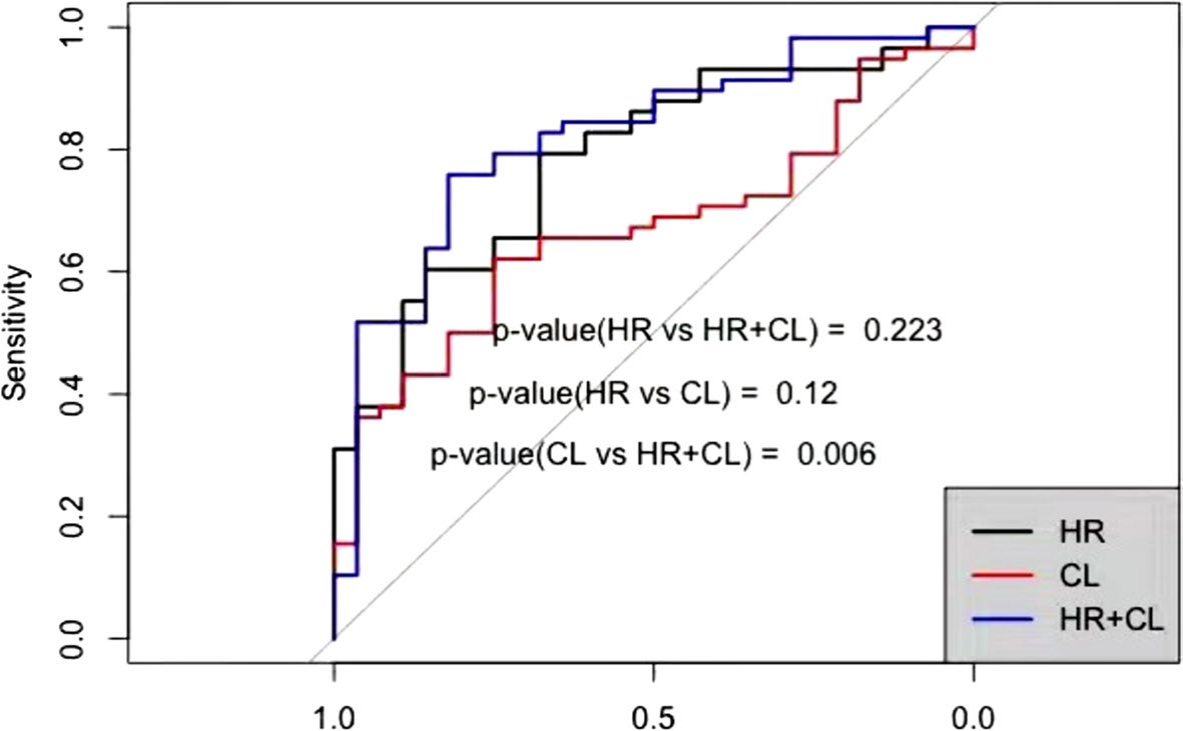
Reciever operating characteristic curves for cervical hardness ratio, cervical length, and hardness ratio combined with cervical length

The AUC of cervical length for the prediction of a successful IOL was 0.671 (95% CI: 0.557–0.785; *P* = 0.010) with a sensitivity of 96.43% and a specificity of 36.21% when 20 mm was used as the cutoff value (Table 4 and Fig. 3). The AUC of HR for the prediction of a successful IOL was 0.785 (95% CI: 0.686–0.884; *P* < 0.001) with a sensitivity of 67.86% and a specificity of 79.31% when a cutoff value of 54.43% was used. The AUC of the combination of cervical length and HR for the prediction of a successful IOL was 0.819 (95% CI: 0.724–0.913; *P* < 0.001) with a sensitivity of 82.14% and a specificity of 75.86%. The predictive value of the combination of cervical length and HR was significantly higher than that of cervical length alone (*P* = 0.006) and not significantly different than that of HR alone (*P* = 0.223). The predictive value of cervical length was not significantly different than that of HR (*P* = 0.120).

**Table 4:** Predictive value of Hardness ratio, cervical length and Hardness ratio combined with cervical length in predicting successful IOL

Parameter	Cut off Value	Sensitivity (%)	Specificity (%)	Positive Likelihood ratio	Negative Likelihood ratio	AUC (95% CI)	*P* value
CL	20.00	96.43	36.21	1.511	0.099	0.671 (0.557–0.785)	0.010
HR	54.43	67.86	79.31	3.280	0.405	0.785 (0.686–0.884)	0.000
HR + CL	38.59	82.14	75.86	3.406	0.234	0.819 (0.724–0.913)	0.000

*AUC* Area under the curve, *CI* Confidence interval, *HR* Hardness ratio, *CL* Cervical length

^*^ *P* < 0.05

## Discussion

Human parturition begins with structural and biochemical changes in the uterus, including changes in the uterine body and cervix. Extracellular matrix changes in collagen organization, water content, and proteoglycan concentration are biomechanical changes that make the cervix soft or hard [12]. As pregnancy progresses, the cervical tissue collagenolytic activity increases [24]. These changes may be identified before the cervix begins to shorten. Cervical ripening plays an important role in inducing labor. In this study, the cervical length and HR measured using the E-cervix system were reproducible and identified as independent predictors of a successful IOL. Furthermore, the predictive value of the combination of cervical length and HR was superior to that of cervical length alone.

The prevalence of unsuccessful IOL varies worldwide. Previous studies have reported that the prevalence of unsuccessful IOL was 17.3% in Hong Kong [23], 24.4% in Northwest Ethiopia [6], and 36.8% in Eastern Ethiopia [7]. In the current study, the prevalence of unsuccessful IOL was 32.56%, which may have been affected by the patients’ cervical parameters. The cervical length was identified as an independent predictor of IOL success. Cervical length decreases as pregnancy progresses [25] and was not traditionally considered to be predictive of a successful vaginal delivery or cervical ripening [26]. The functional cervical length can be measured using the E-Cervix system and a four-point ROI that traces the endocervical lining of a straight or curved cervix [27]. The accuracy and repeatability of cervical length measurements have been improved using semi-automatic systems, and cervical length has become a valuable parameter for predicting the success of IOL [13, 28–31]. Hamza et al. reported that a cervical length of 25 mm, the presence of cervical funneling, and a shorter cervical length were significantly associated with successful IOL within 24 h [28]. Brik et al. reported that patients with a singleton pregnancy who had a longer cervical length prior to induction had an increased risk of unsuccessful IOL [13]. A study conducted by Dagdeviren et al. including 150 nulliparous, full-term, pregnant patients reported that pre-induction cervical length was inversely correlated with vaginal delivery after IOL [29]. Eser et al. reported that cervical length is significantly related to a satisfactory response to IOL and the time from induction to delivery [30]. Zhou et al. found that a short cervical length and a soft cervix are significantly associated with vaginal delivery within 24 h, and that the use of cervical strain elastography could improve the predictive performance of cervical length for a successful vaginal delivery within 24 h of IOL [31].

Dynamic changes of the cervix during labor include softening, dilatation, and effacement. Elastography has been widely used to objectively assess the cervix for maturation. Cervical elastography can be used to predict preterm birth and successful IOL using strain elastography [12, 32]. The quantification of cervical elastography parameters provided by the E-cervix system and cervical strain elastography parameters were obtained by analyzing raw data within a ROI and by describing the HR and mean strain level within 1 cm from the IOS and EOS. Therefore, changes in these parameters may reflect biochemical changes in different areas of the cervix. Collagen and 2PF-active fibrillary structures are significantly related to the mechanical properties of cervical tissue in elastography [33]. The five elastic parameters measured by the E-cervix system provide the strain of different ROIs and characteristics of the cervix, which can be used to detect physiological cervical changes during pregnancy [34]. E-cervix parameters tend to change linearly at term near the time of admission for delivery, and significant differences in E-cervix parameters according to the presence or absence of labor and ECI, IOS, and strain mean values significantly increased as pregnancy progressed [25]. The elastic value of the IOS differs significantly between patients with successful and unsuccessful IOL [23, 35]. Peralta et al. reported that cervical ripening assessed during pregnancy using transient elastography revealed that the EOS was significantly softer than the IOS and that the stiffness values vary significantly in the anterior and posterior cervix [36]. These results are similar to those of the current study. The ECI, EOS, and IOS measured using the E-cervix system were not associated with the success of IOL in this study. The HR was the most valuable of the five E-cervix system parameters and may be used to improve the performance of CL for predicting successful IOL in full-term, nulliparous patients [37]. Zhang et al. reported that HR assessed using the E-cervix system was the most sensitive index for diagnosing cervical insufficiency [19]. In this study, HR was the most accurate indicator of cervical ripening among the five elastic parameters of the cervix included in the study, and it can be used as a predictor of a successful IOL.

The predictive values of cervical strain elastography parameters for successful IOL were compared in this study. The predictive value of HR combined with cervical length was higher than that of cervical length alone, suggesting that HR assessed using the E-cervix system is superior for predicting successful IOL. The cutoff values for cervical length and HR were 20.00 mm and 54.43%, respectively. The predictive performances of HR and cervical length were comparable. In this study, characteristics and cervical strain elastography parameters of patients who underwent successful and unsuccessful IOL were not statistically significant, which is consistent with the results of previous studies [38, 39]. The E-cervix system allows for the use of multiple elastographic images, and the use of the average values of multiple images may reduce the interobserver differences. The E-cervix system monitors the movements around the probe, maintaining consistent conditions during the examination and allowing the acquisition of the strain values and elastographic images only when the strain force is stable. The intraobserver and interobserver reproducibility were moderate to good in this study, suggesting that the HR assessed using the E-cervix system is a reliable predictor of the success of IOL.

### Strength and limitation of this study

This study has a prospective cohort design, which eliminates selection and confounding biases. All patients included in this study followed the same IOL protocol and had a gestational age of 41–41^+6^ weeks, ensuring the comparability of the ultrasound values. However, this study had some limitations. First, this was a single-centered study. Therefore, the patients may not be a representative population. Second, the characteristics of the pregnant women and neonates were not adjusted, which may have affected the rate of successful IOL. Third, data regarding the amount of saline placed in the Cooks cervical ripening balloon were not available. Finally, the predictive model for successful IOL was created for use in this study, and its predictive value was not validated.

## Conclusion

In conclusion, cervical length and HR are predictors of the success of IOL in nulliparous patients during late-term pregnancy. Cervical elastography ultrasound can be used to predict the incidence of successful IOL. The predictive value of the combination of cervical length and HR was higher than that of the cervical length alone. Furthermore, the measurement of elastographic parameters using semiautomatic software has good reproducibility. Therefore, elastographic parameters can be used to predict the success of IOL in nulliparous patients during late-term pregnancy. Additional large-scale, prospective studies should be performed to verify the predictive model, and further research regarding delivery strategies in patients with unsuccessful IOL is necessary.

## Data Availability

The datasets used and/or analyzed during the current study are available from the corresponding author on reasonable request.
